# Combined pentagonal resection and inferior retractor plication in involutional entropion

**DOI:** 10.1186/s12886-018-0986-9

**Published:** 2018-12-19

**Authors:** Janice J. C. Cheung, Carlos K. H. Wong, Leanne T. Y. Cheung

**Affiliations:** 1 Department of Ophthalmology, LKS Faculty of Medicine, The University of Hong Kong, Hong Kong, Hong Kong, Special Administrative Region of China; 2 Department of Ophthalmology, Grantham Hospital, Hong Kong, Hong Kong, Special Administrative Region of China; 3 Department of Family Medicine and Primary Care, LKS Faculty of Medicine, The University of Hong Kong, Hong Kong, Hong Kong, Special Administrative Region of China; 4 LKS Faculty of Medicine, The University of Hong Kong, Hong Kong, Hong Kong, Special Administrative Region of China

**Keywords:** Involutional entropion, Lateral tarsal strip, Inferior retractor plication, Pentagonal resection, Lid laxity, Chinese

## Abstract

**Background:**

To describe the effectiveness of combined pentagonal resection and inferior retractor plication (PR + IRP) based on the Hill’s procedure for the treatment of primary involutional lower eyelid entropion in the Chinese population.

**Methods:**

This is a retrospective review of 52 eyelids of 46 patients who underwent PR + IRP for treatment of involutional entropion between May 2009 and May 2017. Patient demographics, presence of pre-operative laxity, post-operative outcome and presence of complications were all recorded from electronic patient records.

**Results:**

A total of 52 eyelids of 46 patients received PR + IRP. None had recurrence of entropion, 1 (2.1%) had residual entropion, 2 eyelids (4.4%) had lower eyelid notching, 1 eyelid (2.2%) had infection and 1 eyelid (2.2%) had overcorrection. The overall success rate was 90.4%.

**Conclusion:**

Combined PR + IRP is an effective surgical procedure for primary involutional entropion with low recurrence rate. However, it may carry risk of eyelid notching post-operatively.

## Background

Involutional entropion occurs due to aging and can cause irritation to the cornea and patients often present with discharge, discomfort, foreign body sensation and blurred vision in more severe cases. Several mechanisms can either independently, or in combination contribute to its pathogenesis. This includes eyelid horizontal laxity, inferior retractor dehiscence, overriding of the preseptal over the pretarsal orbicularis, weakening of the orbital septum, tarsal plate atrophy and enophthalmos [1–3]. Due to the difference in anatomy of the lower eyelid, Asian eyelids may be more predisposed to involutional entropion than ectropion relative to the Caucasian population [4]. The mechanisms of entropion and its surgical outcomes may therefore also differ.

There are various surgical approaches aiming to correct the underlying mechanism causing entropion, including combined procedures which target multiple mechanisms [5–8]. It is important to determine the contributing factors to the disease pre-operatively. The presence of eyelid laxity should be assessed and documented, this would determine whether eyelid tightening procedures are needed. Lid tightening procedures including lateral tarsal strip (LTS) surgery [5, 9, 10] and wedge resection [11] have been well documented to be effective in the treatment of involutional entropion.

Studies have compared surgical outcome of single procedure with combined procedures and shown a lower recurrence rate after combination procedures [12–14]. Hill et al. [15] previously described a combination technique which involved shortening the lateral lower eyelid by performing a full thickness excision. In addition, a strip of the orbicularis muscle was fixated to the lower tarsal plate border. Curettage of the anterior tarsal plate was carried out to produce a tissue barrier to prevent the overriding of the orbicularis muscle [15]. The Hill’s procedure was subsequently modified and was used in the Caucasian population for recurrent cases with both horizontal and vertical lid laxity [16]. This technique has been further modified at our centre. In our present study, we review the outcomes of the newly modified Hill’s procedure which consists of combined pentagonal resection and inferior retractor plication (PR + IRP) for primary treatment of involutional entropion with lower eyelid laxity in our Chinese population.

## Methods

This was a retrospective study reviewing the medical and surgical records of all patients undergoing entropion corrective surgery between May 2009 and May 2017. Only patients with presence of horizontal and vertical lower eyelid laxity were included. Patients with previous lower eyelid surgery or follow-up less than 6 months were excluded.

The study firmly adhered to the tenets of the 1964 Helsinki declaration and its later amendments. The study protocol was approved by the local institutional research ethics committee.

Electronic medical records were reviewed and patient demographics including age and gender were documented. In addition, preoperative assessment for eyelid laxity, latest follow-up, surgical outcomes and complications including recurrence, residual entropion, over correction, infection, lid contour abnormalities and displacement were also recorded.

### Surgical technique

All surgeries were performed under local anaesthesia with 2% lignocaine in 1:200,000 adrenaline.

Combined PR + IRP was done with subcilliary incision followed by skin flap creation. A strip of orbicularis muscle was identified and cut into two halves vertically. A pentagonal excision of tarsal plate was performed and the grey line, lash line and tarsal plate were approximated with 6-O Vicryl®. The inferior retractor muscle was identified and plicated to the inferior tarsal border with 6-O Vicryl®. Conservative fat excision was done. The strip of orbicularis was shortened and repaired with 6-O Vicryl®. The skin flap was draped over the eyelid and excessive skin was excised. The skin wound was closed.

Success was defined as complete correction of entropion with no recurrence or other complications such as contour abnormalities, residual disease, infection or overcorrection.

## Results

A total of 52 eyelids of 46 patients received PR + IRP. Mean age at surgery was 78.7 years old (range, 52-91 years old). There were 26 male participants and 26 female participants. The right eye was involved in 26 participants and the left eye was involved in 26 participants. The overall mean follow-up time was 20.1 months (range, 6.1–57.1 months) (Table 1).

**Table 1:** Demographics and follow-up time

	PR + IRP
Age at surgery, year mean +/− SD (range)	78.7 +/− 8.7 (52–91)
Sex (male/female), number	26/26
Laterality (R/L), number	26/26
Follow-up, mean months +/− SD (range)	20.1 +/− 17.0 (6.1–57.1)

*SD* standard deviation*, R* Right*, L* Left*, PR Pentagonal resection, IRP Inferior retractor plication*

None of the cases with PR + IRP had any recurrence. One of the cases had overcorrection. The overcorrection was mild, and the patient was asymptomatic, therefore no further surgery performed. There were two cases of post-operative lower eyelid notching. The lower eyelid notching in one of the cases was mild only and asymptomatic, the patient opted for conservative management. The second patient with lower eyelid notching had wound dehiscence and required re-operation with lateral canthotomy, cantholysis and repair of the lower eyelid. According to our definition of success, which is defined by post-operative outcome free of complications and recurrence, the success rate was 90.4% (Table 2).

**Table 2:** Surgical outcome

	PR + IRP
Recurrence, *n* (%)	0 (0.0)
Overcorrection, *n* (%)	1 (2.2)
Residual eyelid laxity, *n* (%)	0 (0.0)
Eyelid notching, *n* (%)	2 (4.4)
Infection, *n* (%)	1 (2.2)
Success rate, *n* (%)	47 (90.4)

*n* number*, PR* Pentagonal resection*, IRP* Inferior retractor plication

## Discussion

In involutional entropion correction surgery, recurrence is the most important complication. Studies comparing simple surgical procedures with combined procedures have shown combined procedures tend to have lower recurrence rate [12, 17–19]. In our study, we describe a new approach based on Hill’s procedure in involutional entropion with documented eyelid laxity in the Chinese population. LTS is a popular procedure for eyelid shortening and can be used either in entropion or ectropion correction [7, 9]. Although LTS is effective in tightening the eyelid, it is known to have disadvantages such as under correction, webbing, recurrence and disruption of the lateral canthal angle [20, 21]. López-García et al. [7] reported recurrence rate of 17.4% after conventional tarsal strip surgery for involutional entropion. It can be combined with other procedures such as everting sutures [13, 14, 17] and inferior retractor plication, shortening or advancement [12, 18, 19]. There is a range of recurrence rates with such combined procedures. Rabinovich et al. [22] reported zero recurrence rate using LTS with infraciliary rotation sutures at 1–67 months follow up. However, cases with less than 6 months follow-up were also included in their study. The combined procedure including LTS, inferior retractor tightening and everting sutures described by Serin et al. had 2.2% recurrence rate [12]. Ho et al. [14] reported a recurrence rate of 9.4% in primary cases and 22.2% after failed previous procedure after lateral tarsal strip and Quickert sutures for lower eyelid entropion. Allen et al. [23] described a modified Quickert and Jones technique which included wedge resection of the eyelid. They similarly reported a recurrence rate of 0% but experienced complications such as ectropion and eyelid notching.

The technique of PR + IRP is based on the Hill’s procedure [15] which has already been previously modified [16]. The previous modification shortened the lower eyelid with a central full thickness resection instead of at the lateral aspect of the lower eyelid. Other modifications included creation of a skin flap, the omission of curettage of the anterior tarsal surface and suturing the orbicularis muscle to the tarsal plate border [16]. In our present study, this technique was further modified to omit fixation of the orbicularis muscle to the tarsal border and omit the use of tractions sutures. Previously, traction sutures were taped to the forehead and maintained for two to three days in the modified Hill’s technique. It was found that with such modification the recurrence rate is still low. However, we did encounter two cases with eyelid notching post-operatively. Although it was only mild in one of the cases, the other patient had wound dehiscence and required re-operation. This may be related to excessive wound tension after approximation of the lower eyelid after resection.

In our study, the recurrence rate of PR + IRP was zero. It has been reported that the Asian lower eyelid anatomy is different from Non-Asian lower eyelids [24, 25]. In particular, the protrusion of the orbital fat pad may contribute to the mechanism of lower eyelid entropion. In the PR + IRP technique, an important step other than shortening the eyelid and plicating the inferior retractor, is addressing the orbital fat pad, orbicularis oculi muscle and skin which also produces a vertical vector in preventing internal rotation. The herniated fat is excised, and the orbicularis muscle shortened, preventing further fat protrusion. This may explain its effectiveness in the Asian population.

Although none of the cases encountered recurrence, some cases did have complications such as overcorrection, eyelid notching and infection. However, this was only mild in most cases and only one case required re-operation for eyelid notching.

Our study is limited by its retrospective nature and shares all the limitations of a retrospective study. The modification of Hill’s procedure using the pentagonal block resection and inferior retractor plication technique has a low recurrence rate and the outcomes are satisfactory. Regardless of the approach, the surgeon may want to ensure that the fat pad, orbicularis oculi muscle and skin are appropriately addressed, especially in the Chinese population.

## Conclusion

The combined PR + IRP technique is effective in correcting involutional entropion with horizontal eyelid laxity in the Chinese population and is associated with high success rate and low recurrence rate.

## Abbreviations

IRP: Inferior retractor plication
LTS: Lateral tarsal strip
PR: Pentagonal resection
